# Nucleolar localization of the Notch4 intracellular domain underpins its regulation of the cellular response to genotoxic stressors

**DOI:** 10.1038/s41420-020-0242-y

**Published:** 2020-02-18

**Authors:** Neetu Saini, Apurva Sarin

**Affiliations:** 1 Institute for Stem Cell Science & Regenerative Medicine (inStem), Bellary Road, Bengaluru, Karnataka India; 2 Department of Biology, Manipal Academy of Higher Education, Manipal, India

**Keywords:** Apoptosis, Breast cancer

## Abstract

Cell survival is one of the many cellular processes regulated by Notch family of proteins. A comparison of human breast cancer cell lines, which differ in the levels of endogenous Notch4, implicated the protein in regulating susceptibility to apoptosis triggered by genomic damage. In agreement with this observation, increased susceptibility to genotoxic damage was observed following siRNA ablations of Notch4 in two breast cancer cell lines. Further, overexpressing Notch4 intracellular domain (NIC4) tagged to GFP (NIC4-GFP), protected cells from apoptosis triggered by genotoxic drugs. In cells immune-stained for endogenous Notch4, protein was detected in the nucleolus and nucleoplasm, which was also confirmed by the co-localization of NIC4-GFP with RFP-tagged nucleolar proteins in breast cancer cells or the unrelated HEK cell line. Linking functional outcomes to nucleolar localization, NIC4-GFP protection from apoptosis, required the nucleolar proteins Nucleolin and Fibrillarin. Consistently, immunoprecipitation analysis revealed associations between nucleolar proteins—Nucleolin and Nucleophosmin—and Notch4. Microscopy-based biophysical analysis of live cells showed that nucleolar and nucleoplasmic pools of NIC4-GFP are mobile, with some sequestration of nucleolar NIC4-GFP pools. A nucleolar excluded form, NIC4_3RA-GFP, generated by site-directed mutagenesis of the nucleolar localization sequence in NIC4, could not protect from apoptosis triggered by genotoxic stressors. However, transcriptional activity or protection from apoptosis triggered by endoplasmic stress was comparable in cells expressing NIC4_3RA-GFP or NIC4-GFP. Together, the data show that nucleolar localization of NIC4 is critical for the regulation of genomic damage and may be uncoupled from its activities in the nucleoplasm. This study identifies intrinsic features of NIC4 that regulate signaling outcomes activated by the receptor by controlling its spatial localization.

## Introduction

Signaling through Notch receptors protects cells from varied apoptotic stimuli^1–4^. Four Notch receptors (Notch1–4), thought to be activated by binding to one of the five ligands, Delta-like 1/3/4 or Jagged 1/2, are known in mammals^5,6^. Notch receptors comprise an extracellular domain with multiple epidermal growth factor-like repeats, a transmembrane domain, and an intracellular domain^7^. Ligand-activated Notch undergoes a series of proteolytic cleavages, releasing the intracellular domain (Notch4 intracellular domain (NIC), which generally localizes to the nucleus^8–10^ and complexes with cofactors recombination signal–binding protein-Jκ (RBPj-κ) and Mastermind like, to induce transcription of various genes^11,12^. Apart from this core canonical pathway, there are several reports of atypical ligand-independent and non-nuclear Notch signaling in diverse systems^13–17^.

Notch4 signaling promotes breast cancer cell survival in response to diverse treatment modalities^18–20^ and is implicated in poor prognosis and high risk of relapse in breast cancer patients^21^. Notch4 signaling regulates susceptibility to cell death in human B cell acute lymphoblastic leukemia^1^, endothelial^3^, as well as cells of pancreatic origin^22^ among others. Hence, while many studies have correlated Notch4 signaling with resistance to cell death, in-depth analysis of molecular details underlying Notch4-mediated anti-apoptotic activity have not been undertaken.

Here we characterize a signaling cascade arising from the nucleolar localization of NIC4 that confers protection from genomic damage in mammalian cells. We demonstrate functional and biochemical interactions with nucleolar proteins as well as on cellular machinery that senses and repairs genomic damage. Despite the dynamic nature of nuclear and nucleolar NIC4-GFP (green fluorescent protein) revealed in biophysical assays in live cells, we find that protection from genomic damage is spatially restricted and controlled by the nucleolar localization sequence (NoLS) in the NIC4. The experiments also reveal that nuclear and nucleolar functions of NIC4 can be uncoupled. Taken together, we identify a key role for nucleolar localization of NIC4 that underlies the integration with signaling cascade activated in response to and confers protection from genomic damage.

## Results

### Notch4 regulates cellular susceptibility to genomic damage

**Fig. 1:**
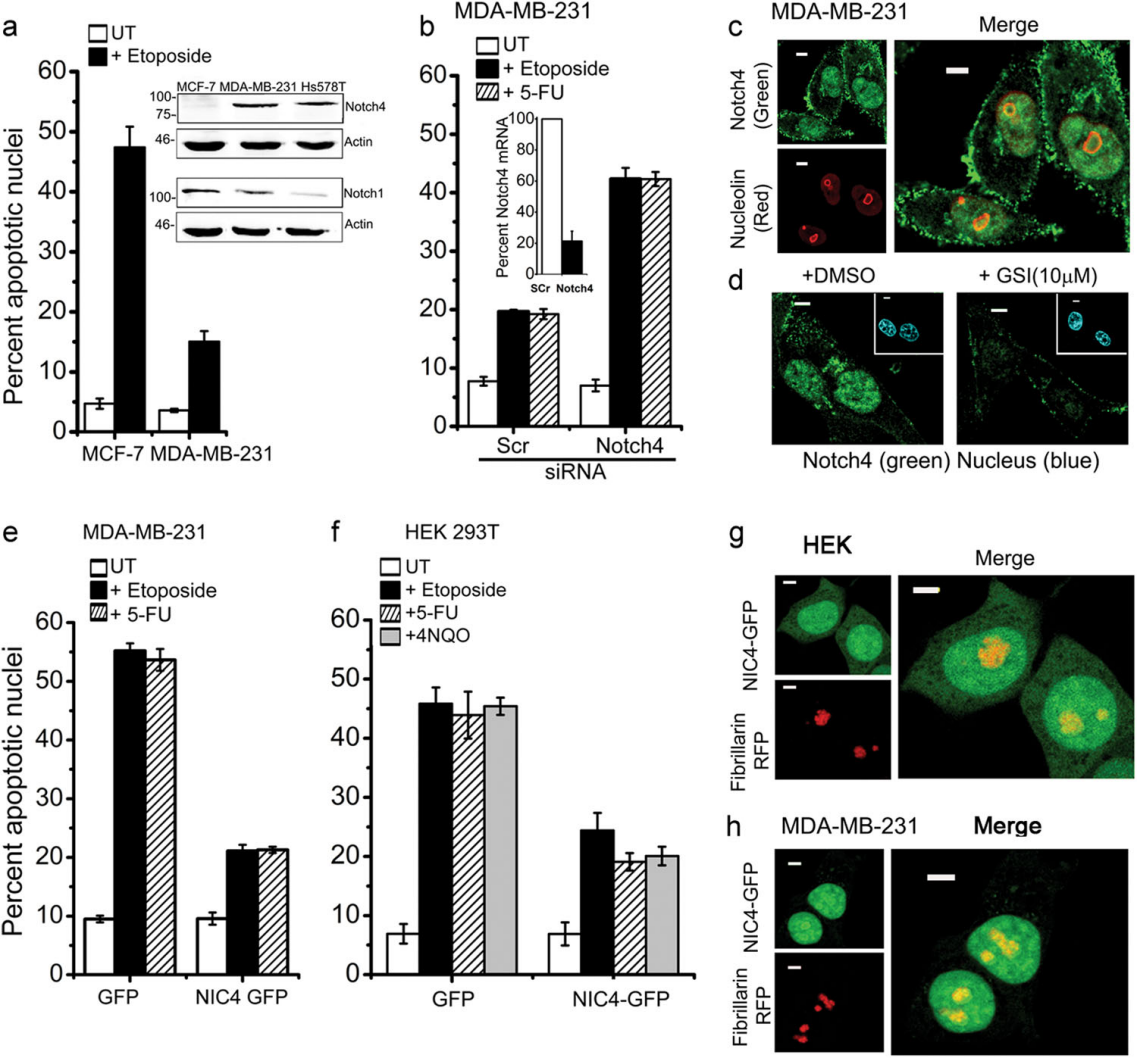
Notch4 protects from apoptosis triggered by genotoxic agents. **a** Apoptotic nuclear damage in MCF-7 and MDA-MB-231 cell lines continued untreated or treated with 10 μM etoposide for 24 h in serum-free medium. Inset: immunoblot of cell lysates probed for Notch4 and Notch1 proteins. **b** Apoptotic damage in MDA-MB-231 cells pretreated with siRNA to Notch4 or a scrambled control for 48 h and then continued untreated (UT) or treated with etoposide (10 μM) or 5-FU (10 μM) for 24 h in serum-free medium. Inset: percentage of Notch4 transcripts in the siRNA-treated groups. **c** Representative confocal images of cells stained for Notch4 (green) and Nucleolin (red) as described in “Methods” (Manders correlation coefficient: 0.73 ± 0.23). **d** Representative confocal images of cells stained for Notch4 in cells pretreated with the vehicle or GSI-X (10 μM) for 24 h in serum-free medium. **e** Apoptotic nuclear damage in cells expressing GFP or NIC4-GFP and treated with etoposide or 5-FU for 48 h in serum-free medium. **f** Percentage of apoptotic nuclear damage in HEK cells expressing GFP or NIC4-GFP and treated with etoposide or 5-FU or 4NQO (5 μM) for 48 h in serum-free medium. **g** Representative confocal images of HEK cells co-expressing NIC4-GFP and Fibrillarin-RFP imaged 24 h after transfection (Manders correlation coefficient: 0.87 ± 0.15). **h** Representative confocal images of MDA-MB-231 cells co-expressing NIC4-GFP and FBL-RFP imaged 24 h after transfection (Manders correlation coefficient: 0.96 ± 0.07). Apoptotic nuclear damage was scored by visualizing nuclei stained with Hoechst 33342. Data represent the mean ± S.D. of three independent experiments. Scale bar: 5 μm.

Chemicals such as etoposide trigger genomic damage culminating in apoptosis in mammalian cells^23,24^. We observed that the MCF7 and MDA-MB-231 cell lines differ in susceptibility to apoptosis triggered by etoposide, that is, 24 h after treatment, MCF7 cells show significant induction of apoptotic damage, whereas the levels of damage are relatively low (10–20%) in MDA-MB-231 cells (Fig. 1a). Further, it was also observed that the two cell lines differ in the levels of Notch4 protein (Fig. 1a inset and Supplementary Fig. 1A). To assess whether Notch4 confers protection from cell death, RNAi-mediated ablations of Notch4 in MDA-MB-231 cells were undertaken. Cells pretreated with small interfering RNA (siRNA) to Notch4, in contrast to cells treated with a scrambled control, showed increased sensitivity to apoptosis, following treatment with the DNA-damaging agents etoposide or 5-fluorouracil (5-FU) for 24 h (Fig. 1b). Since other studies have shown that the spatial distribution of Notch family proteins underpins signaling outcomes in diverse contexts^13,16,17^, MDA-MB-231 cells were immune-stained to assess Notch4 localization. Intriguingly, subcellular distribution visualized by confocal microscopy in cells stained with an antibody to Notch4 revealed discrete areas of intense staining as well as a generalized or diffuse distribution in the nucleus (green, Fig. 1c) and at the cell membrane (Fig. 1c and Supplementary Fig. 1B). The brighter intense spots in the nucleus overlapped with staining for Nucleolin (NCL, red, Fig. 1c), indicating co-localization at the nucleolus^25^. Nuclear Notch4 staining was lost in cells treated with a gamma-secretase inhibitor (GSI)-X, which blocks Notch processing and release of NIC (Fig. 1d and Supplementary Fig. 1C), indicating that the antibody detects processed Notch4 (NIC4). Residual non-nuclear, GSI-X-resistant staining most likely represents full-length Notch4.

We next tested whether overexpression of GFP-tagged processed intracellular domain of Notch4 (NIC4-GFP) recapitulated these observations. MDA-MB-231 cells expressing NIC4-GFP were protected from apoptosis triggered by etoposide or 5-FU (Fig. 1e). Similarly, HEK cells expressing NIC4-GFP were protected from apoptosis triggered by etoposide, 5-FU (Fig. 1f) or following treatment with the quinoline compound 4-nitroquinoline *N*-oxide (4NQO) (Fig. 1f), which mimics radiation-induced DNA damage^26^. As seen for endogenous NIC4, NIC4-GFP was distributed in the nucleoplasm and nucleolus and co-localized with co-transfected Fibrillarin (FBL)-RFP (red fluorescent protein) in HEK cells (Fig. 1g and Supplementary Fig. 1D). This distribution was reproduced in MDA-MB-231 cells overexpressing NIC4-GFP, which matched the pattern of localization of endogenous NIC4 (Fig. 1h and Supplementary Fig. 1E). These experiments established that nucleolar localization is a feature of endogenous and overexpressed NIC4. Molecular complexes that coordinate the cellular response to DNA damage are localized at the nucleolus^27,28^. In order to molecularly characterize the pathway, we assessed whether NIC4-mediated signaling required nucleolar proteins or molecules regulating DNA repair.

### Dependence on nucleolar proteins for anti-apoptotic activity

**Fig. 2:**
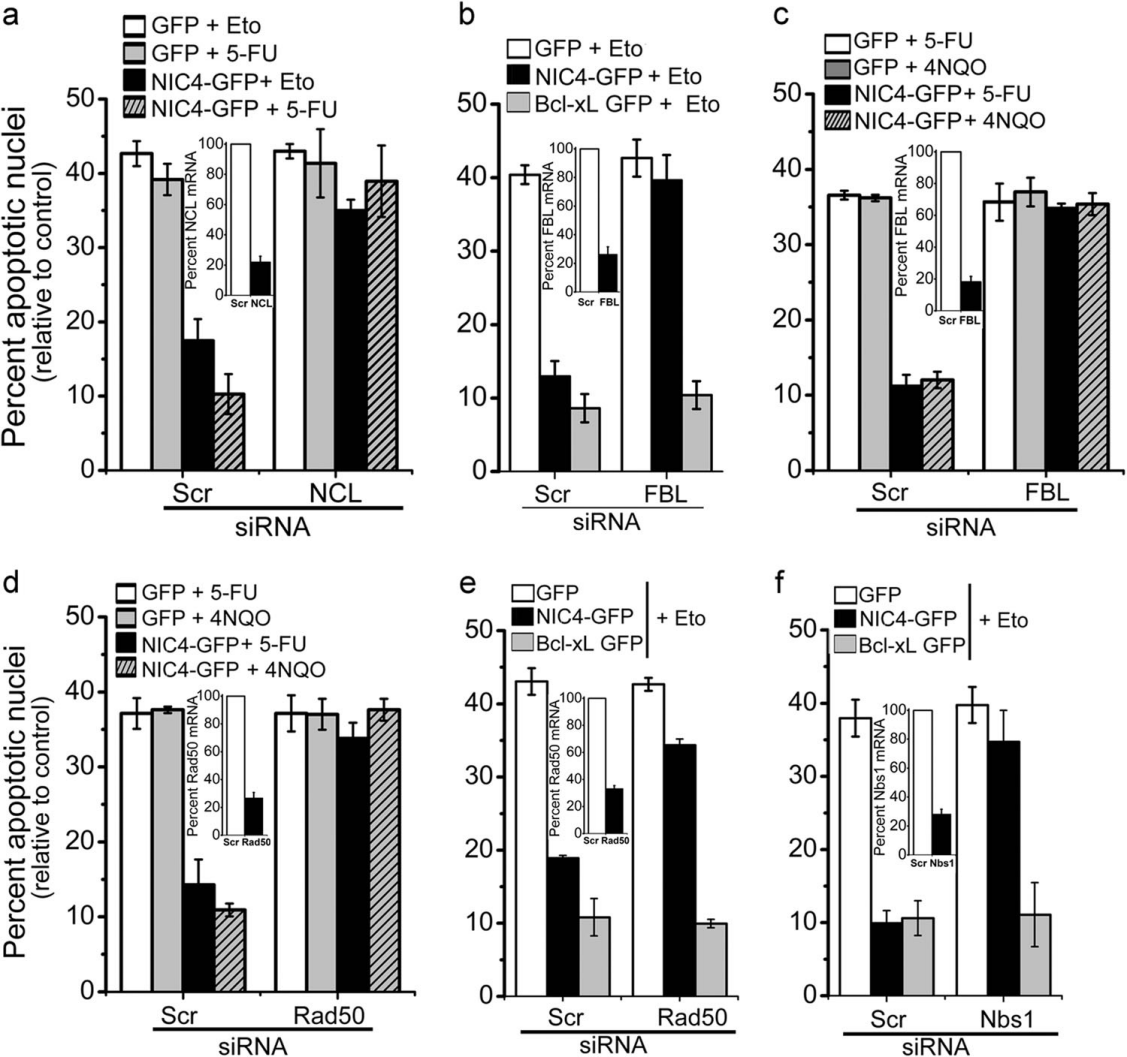
Nucleolar proteins and the DNA damage response proteins are required for NIC4-mediated anti-apoptotic activity. **a**–**f** Apoptotic nuclear damage in HEK cells pretreated with siRNA shown in each panel, transfected with plasmids as shown and treated with 10 μM etoposide or 10 μM 5-FU or 5 μM 4NQO for 48 h in serum-free medium. Insets: percentage of mRNA levels in the transfected groups. Data are mean ± S.D. of three independent experiments.

NIC4-mediated anti-apoptotic activity was assessed in HEK cells pretreated with siRNA targeting the proteins NCL or FBL, which are predominantly nucleolar localized. Ablation of either protein abrogated NIC4-mediated protection from apoptosis triggered by etoposide, 5-FU or 4NQO (Fig. 2a–c). Consistently, the ablation of NCL or FBL in MDA-MB-231 cells increased susceptibility to apoptosis (Supplementary Fig. 2A), additional evidence that nucleolar intermediates are required for cellular repair and recovery from genomic damage. Further, in agreement with other reports, NCL overexpression in HEK cells also protects cells from apoptosis via a pathway depending on DNA repair intermediates (Supplementary Fig. 2B). Suggesting a hierarchy specific to NIC4, neither NCL nor FBL was required for NIC1 (Supplementary Fig. 2C) or Bcl-xL (B cell lymphoma extra-large; a BCl-2 family anti-apoptotic protein) mediated protection from genomic damage (Fig. 2b). Furthermore, the canonical nuclear partner RBPj-κ was dispensable for NIC4-mediated anti-apoptotic activity (Supplementary Fig. 2D). Together, these experiments reveal that NIC4 activated a signaling cascade involving nucleolar intermediates, some of which themselves have demonstrable capabilities to mitigate genomic damage. Given the unusual features of NIC4 signaling uncovered by these experiments, we next assessed dependence, if any, on canonical intermediates of DNA repair.

The MRN (Mre11/Rad50/Nbs1) sensor complex comprises the Mre11–Rad50–Nbs1 proteins that act in concert to sense DNA damage and initiate repair^29–31^. Employing siRNA-mediated ablations, we tested the requirement for Nbs1 (Nijmegen Breakage Syndrome 1) and Rad50 for NIC4-mediated protection from genomic damage. Depletion of Rad50 abrogated NIC4-mediated inhibition of 4NQO, 5-FU, or etoposide-induced apoptosis (Fig. 2d, e) but was dispensable for Bcl-xL-mediated anti-apoptotic activity (Fig. 2e). Similarly, Nbs1 is required for NIC4-mediated activity and is dispensable for Bcl-xL-mediated protection (Fig. 2f). Thus NIC4 signaling integrates with molecular complexes that sense and repair genomic damage, as well as nucleolar-resident proteins—FBL and NCL—in protection from genomic damage.

### Notch4 localization and associations within the nucleolus in breast cancer cell lines

**Fig. 3:**
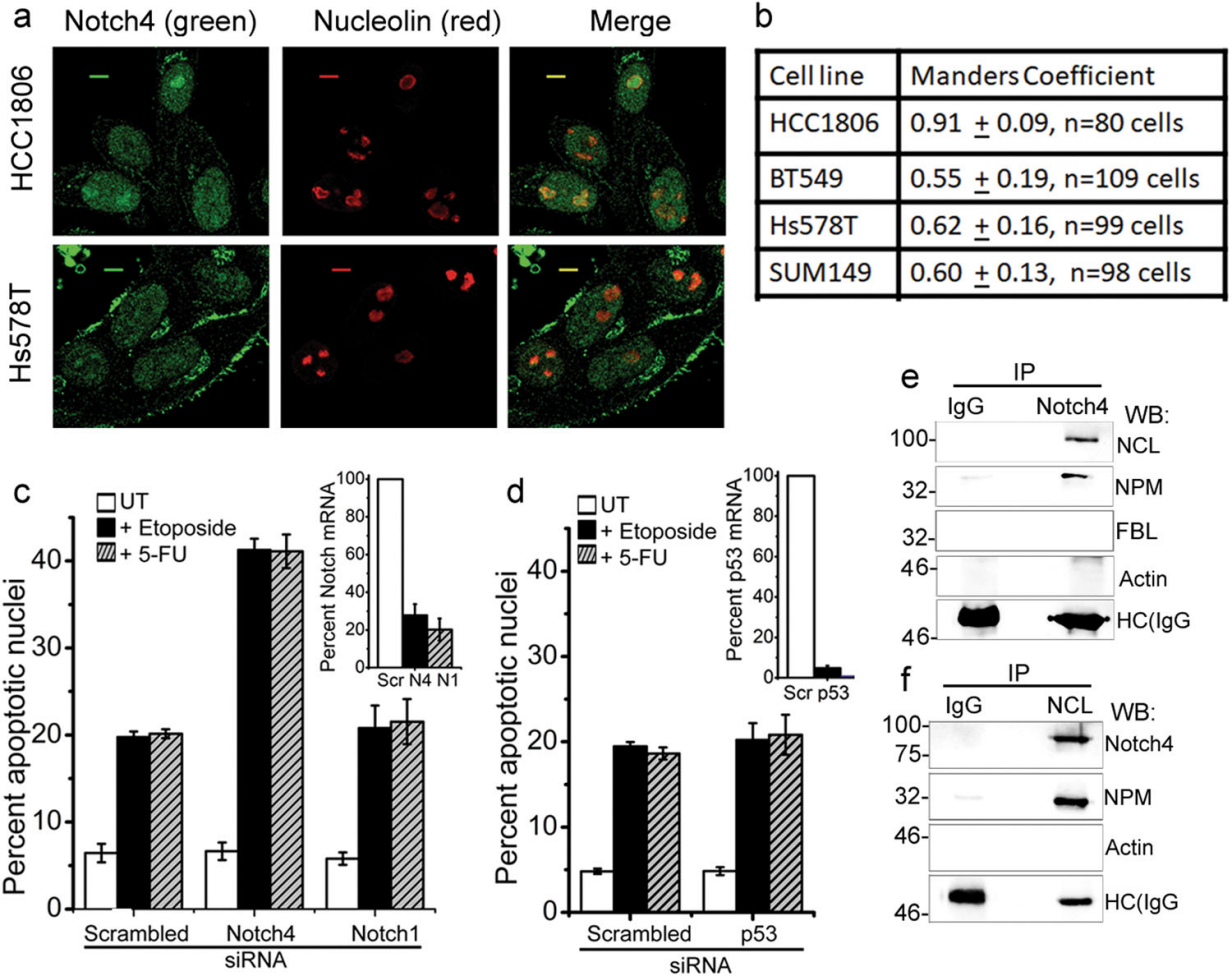
Notch4 localization in breast cancer cell lines. **a** Representative confocal images of cell lines stained for Notch4 (green) or Nucleolin (red) and merged images. **b** Co-localization of Nucleolin (red) and Notch4 (green), quantified by Manders Coefficient (described in “Methods”) for the indicated cell lines. **c**, **d** Apoptotic nuclear damage in Hs578T cells pretreated with the indicated siRNA for 48 h and then cultured untreated (UT) or with 10 μM etoposide or 10 μM 5-FU for 24 h. Inset: percentage of mRNA levels in siRNA-transfected groups. **e**, **f** MDA-MB-231 cells treated with etoposide for 6 h were lysed and subjected to immunoprecipitation with antibody to Notch4 (**e**) or NCL (**f**) and IgG (isotype control). Immunoprecipitates were analyzed by western blotting for NCL, Notch4, NPM, FBL, Actin, and IgG. Data show the mean ± S.D. of three independent experiments. Scale bar: 5 μm.

Next, the localization of endogenous Notch4 was assessed in HCC1086, BT-549, Hs578T, and SUM149 breast cancer cell lines. Notch4 staining was detected in the nucleoplasm and co-localized with endogenous NCL in all cell lines (Fig. 3a, b and Supplementary Fig. 3A–E). This is in striking contrast and distinct from the distribution of the closely related protein, Notch1, wherein the processed receptor albeit nuclear localized is excluded from the nucleolus (Supplementary Fig. 3F–H). Further, siRNA-mediated silencing of Notch4 increased sensitivity of Hs578T cells to etoposide or 5-FU-mediated apoptosis (Fig. 3c), which is p53 dependent in these cells (Fig. 3d). Ablation of Notch1 was without effect in this context (Fig. 3c).

Next, direct associations of NIC4 with nucleolar proteins were assessed by immunoprecipitation analysis. Complexes precipitated with an antibody to Notch4 but not IgG (isotype control) included NCL as well as its known interactor Nucleophosmin (NPM; Fig. 3e). FBL was excluded from this complex, suggesting stable direct associations of NIC4 with a subset of nucleolar proteins. Consistently, in reverse immunoprecipitations, immune-complexes precipitated by antibody to NCL or NPM independently confirmed inclusion of NIC4 in these complexes (Fig. 3f and Supplementary Fig. 3I).

In order to explore the relationship between the nuclear and nucleolar pools, the subsequent experiments examined the dynamics underlying the subcellular distribution of NIC4.

### Dynamics of NIC4 localization to the nucleolus

**Fig. 4:**
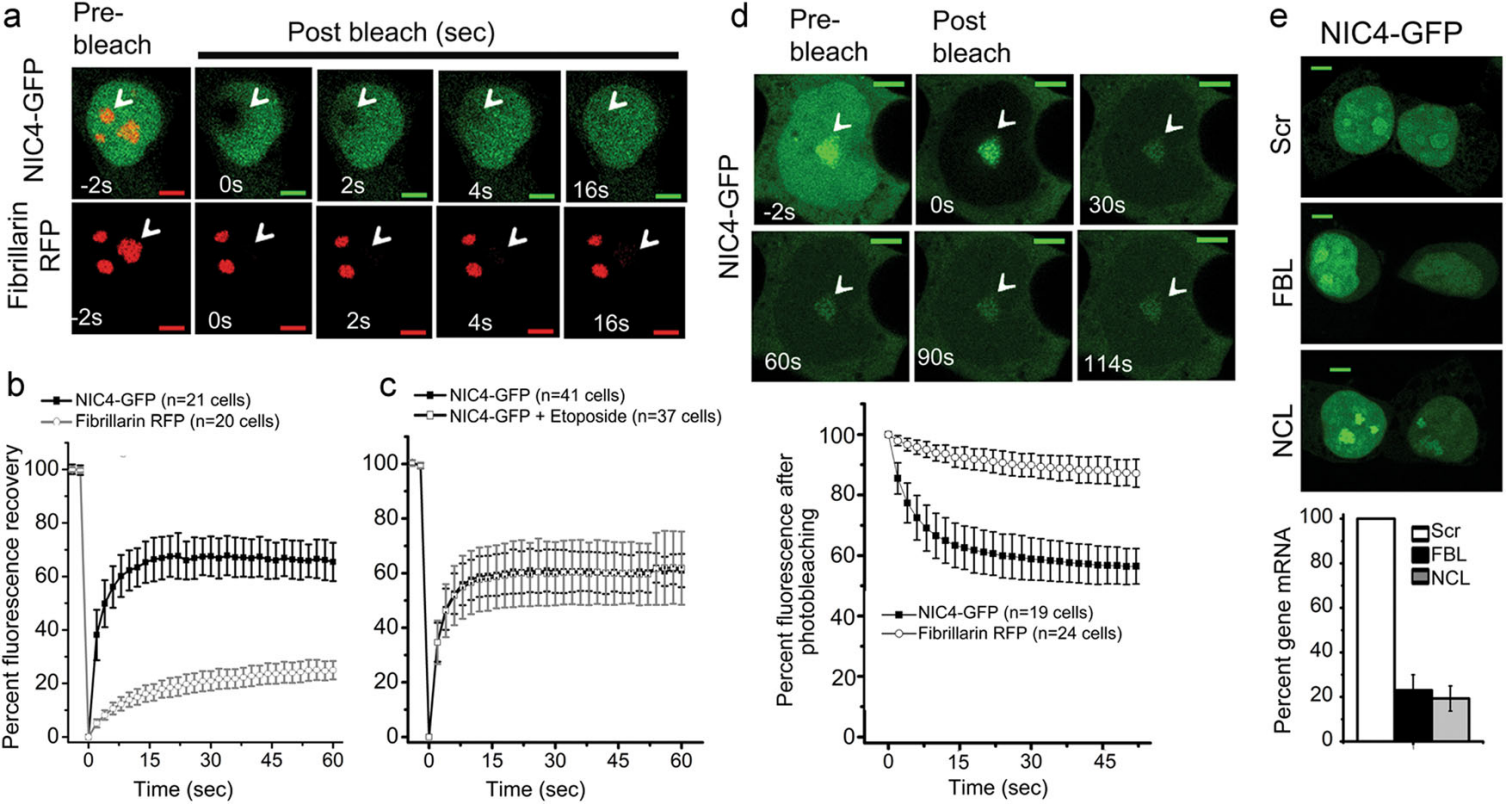
Cellular dynamics of NIC4-GFP. **a** Representative confocal images of a cell co-expressing FBL-RFP and NIC4-GFP. The white arrowhead indicates a bleached nucleolus. **b**, **c** Fluorescence intensity recovered over time following photo-bleaching of cells co-expressing FBL-RFP and NIC4-GFP (**b**) and treated for 6 h with 10 μM etoposide (**c**). Data plotted are mean ± SD of 20 cells in **b** and 37 cells in **c**. **d** Loss of fluorescence intensity over time (FLIP) in cells co-expressing FBL-RFP and NIC4-GFP. The panel shows a cell over time, with the visualization spot (not bleached) marked by an arrow-head, surrounded by the bleached (dark) region. Change in fluorescence over time is plotted in the graph below. Data plotted are mean ± S.D. of a minimum of 19 cells in each condition. **e** Representative confocal images of HEK cells expressing NIC4-GFP following treatment with siRNA shown. mRNA levels of the indicated transcripts in siRNA-treated cells is plotted. Images are representative of 30 cells in each condition. Scale bar: 5 μm.

Since the previous experiments established that ectopically expressed protein recapitulated the pattern of distribution of endogenous protein, we characterized dynamics by fluorescence recovery after photo-bleaching (FRAP) analysis in live cells, co-expressing NIC4-GFP and FBL-RFP. In this analysis, following a bleach of one spot, which marked nucleolar-localized NIC4-GFP, the recovery of GFP fluorescence was rapid with approximately 60% of the original intensity restored within few seconds post photo-bleaching (Fig. 4a, b and Supplementary Fig. S4A). NIC4-GFP dynamics were not accelerated or reduced in cells treated with etoposide 6 h (Fig. 4c and Supplementary Fig. 4B). Hence, while a large proportion of NIC4-GFP moves freely between the nucleoplasm and the nucleolus, a fraction of nucleolar NIC4-GFP has restricted mobility. Expectedly, the recovery of Fibrillarin-RFP fluorescence following photo-bleaching is low (Fig. 4a, b and Supplementary Fig. 4A).

The mobility of nucleolar pools of NIC4-GFP was also assessed by fluorescence loss in photo-bleaching (FLIP) analysis in live cells. In this assay, nucleoplasm was photo-bleached and changes in fluorescence, if any, in the nucleolus were tracked over time. If NIC4 is freely diffusible, diminished fluorescence in the nucleolus is expected, following the bleach of the surrounding region. NIC4-GFP fluorescence in the nucleolus is reduced by ~40%, relative to the intensity at the onset of the assay and did not diminish further with time (Fig. 4d and inset images and Supplementary Fig. 4C). This is consistent with the immobile fraction of nucleolar NIC4-GFP detected in the FRAP analysis. The loss of fluorescence of FBL-RFP was minimal and protein was not detected in regions outside the nucleolus (Fig. 4d and Supplementary Fig. 4c). Further, the subcellular distribution of NIC4 was unchanged in cells ablated for either NCL or FBL (Fig. 4e and Supplementary Fig. 4D). While the experiments thus far have indicated interactions with nucleolar proteins, subsequent experiments were designed to test whether localization to the nucleolus was required for NIC4 activity.

### Nucleolar localization regulates NIC4 signaling

**Fig. 5:**
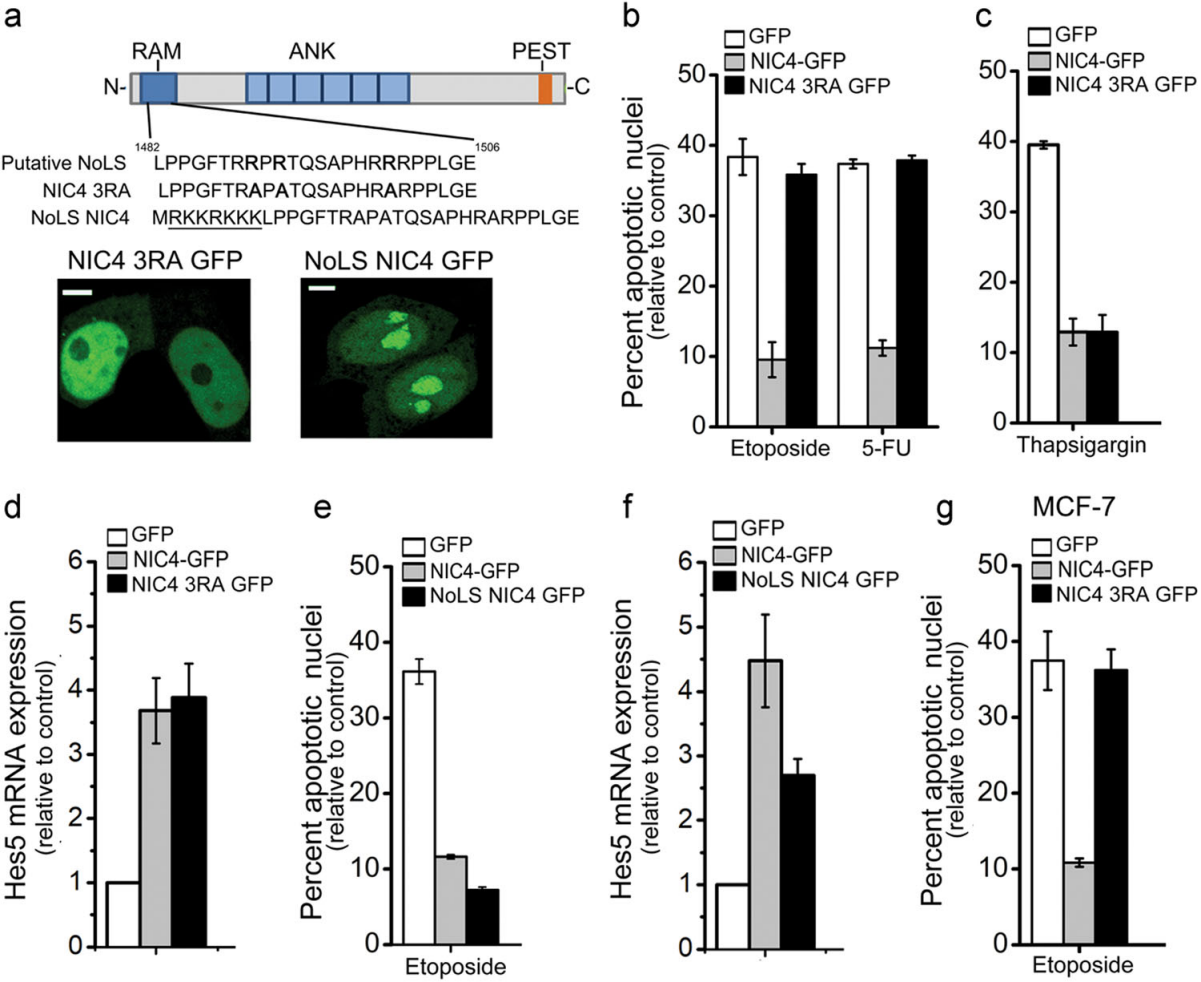
Nucleolar localization of NIC4 is required for protection from genomic stress. **a** Schematic showing the putative NoLS sequence in NIC4, the modifications in NIC4_3RA (with three R residues replaced by A in the NoLS), and the additional NoLS at the NIC4 N-terminal. Representative confocal images of HEK cells expressing NIC4_3RA-GFP (left) and NoLS_NIC4 GFP (right). Scale bar: 5 μm. **b**, **c** Apoptotic nuclear damage in cells expressing GFP, NIC4-GFP, or NIC4_3RA-GFP, treated with 10 μM etoposide or 10 μM 5-FU for 48 h (**b**) or 10 μM Thapsigargin for 20 h (**c**). **d** Relative transcript levels of Hes5 in cells transfected with GFP, NIC4-GFP, or NIC4_3RA-GFP and cultured for 36 h in complete medium. **e** Apoptotic nuclear damage in cells expressing GFP, NIC4-GFP, or NoLS_NIC4-GFP, treated with etoposide for 48 h in serum-free medium. **f** Relative transcript levels of Hes5 in cells transfected with the indicated plasmids and cultured for 36 h. **g** Apoptotic nuclear damage in MCF7 cells expressing GFP, NIC4-GFP, or NoLS_NIC4-GFP, treated with etoposide for 48 h in serum-free medium. Data plotted are mean ± S.D. of three independent experiments.

Modified forms of NIC4 were generated that were targeted to the nucleolus by the addition of addressing tags or prevented from nucleolar localization by side-directed mutagenesis and analyzed for regulation of apoptosis as well as conventional transcriptional outcomes. NIC4 was tagged with an additional NoLS derived from the nuclear factor-κB-inducing kinase^32^, and in order to disrupt nucleolar localization^33^, the positively charged Arginine residues 1490, 1492, and 1501—in the putative NoLS of NIC4—were replaced by the neutral amino acid Alanine (Fig. 5a). The subcellular localization of NoLS_NIC4-GFP and NIC4_3RA-GFP expressed in HEK cells was visualized by confocal microscopy. Expectedly, NIC4_3RA-GFP was excluded from the nucleolus and localized to the nucleoplasm (Fig. 5a and Supplementary Fig. 5A). NoLS_NIC4-GFP was enriched in the nucleolus, with very low levels of GFP signal detected in the nucleoplasm (Fig. 5a and Supplementary Fig. 5B).

**Fig. 6:**
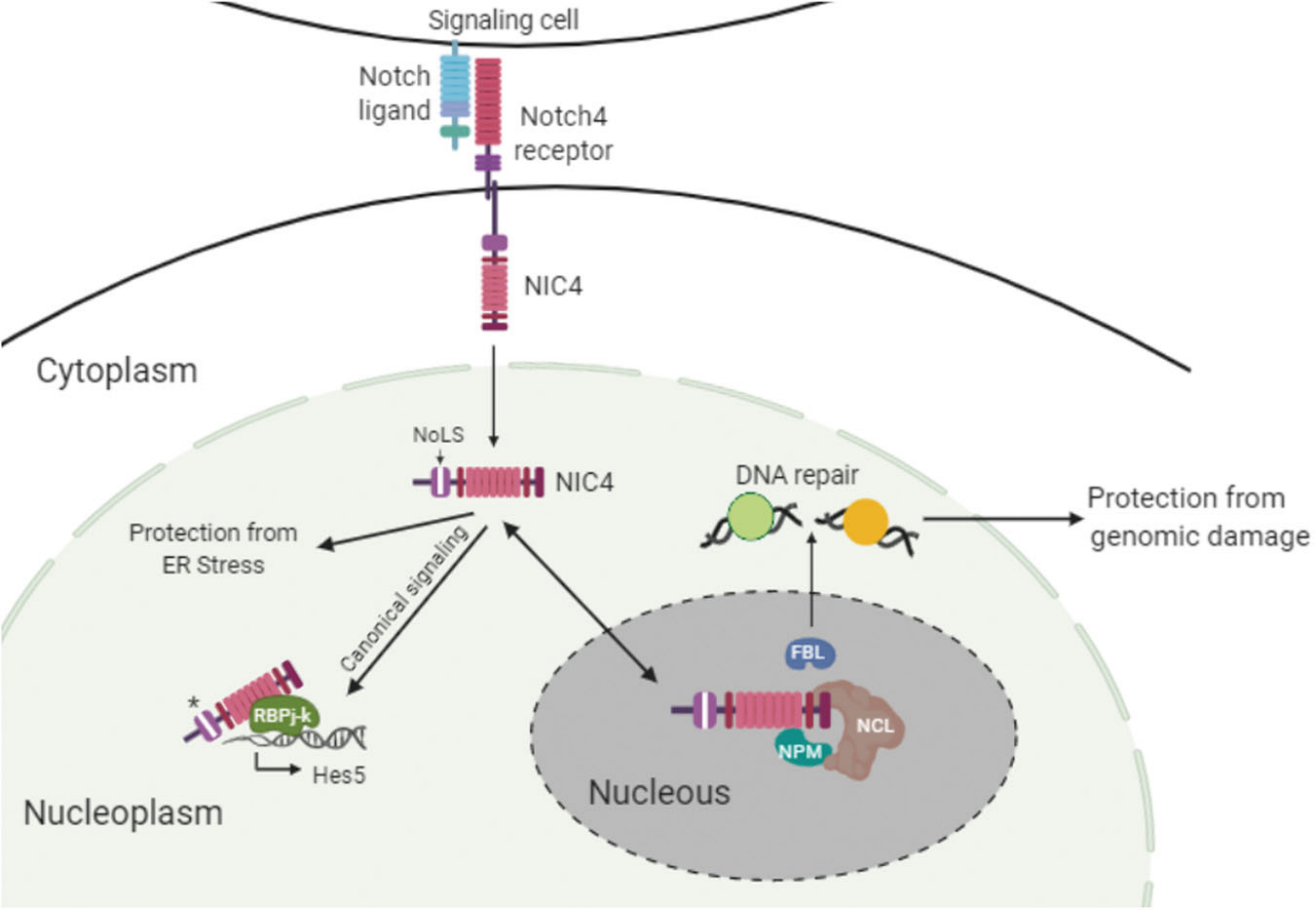
Schematic summarizing key observations of the current study. NIC4 mobility in the nucleus and nucleolus is dynamic with NIC4 localization to the nucleolus, guided by its NoLS. NIC4 associates with NCL and NPM, and protection from genomic stressors is dependent on nucleolar proteins NCL and FBL. Nucleolar functions can be uncoupled from nuclear activities of NIC4. Not to scale.

Consistent with the dependence on and the association with nucleolar proteins, NIC4_3RA-GFP did not protect from genomic damage (Fig. 5b). However, NIC4_3RA-GFP protection from Thapsigargin (an endoplasmic reticulum (ER) stressor) induced apoptosis was comparable to NIC4-GFP-expressing cells (Fig. 5c), reinforcing the importance of its nucleolar localization for NIC4-mediated protection from genomic damage. Further, the induction of Hes5 (hairy and enhancer of split-5) transcripts, a readout of a canonical function, was comparable in both NIC4- and NIC4_3RA-GFP-transfected groups (Fig. 5d), establishing that NIC4_3RA retains transcriptional activity. In related experiments, inhibition of genomic damage by NoLS_NIC4-GFP was comparable to NIC4 (Fig. 5e), whereas Hes5 induction was (expectedly) attenuated in cells expressing NoLS_NIC4-GFP (Fig. 5f). Finally, we show that expression of NIC4-GFP but not NIC4_3RA-GFP protected MCF7 cells from apoptosis (Fig. 5g). In summary, nucleolar pools of NIC4 act in conjunction with nucleolar-resident proteins to confer protection from apoptotic damage triggered by genomic stressors. This outcome is uncoupled from NIC4 signaling in the nucleoplasm (Fig. 6).

## Discussion

Notch family proteins integrate with a range of signaling cascades that underlie pleiotropic outcomes of Notch signaling^15–17^. Here we provide evidence for spatial regulation of Notch4 signaling and its integration with a signaling cascade mediating protection from genomic damage. To our knowledge, nucleolar occurrence of NIC4 controlled by a functional NoLS and the consequence to signaling is a newly described feature of the pathway, detailed in this report.

Functional and biochemical analysis demonstrating the interactions and dependence on nucleolar proteins was confirmed by imaging analysis as well as by measurements of NIC4 dynamics in live cell nuclei. siRNA-based ablations identified the requirement for the nucleolar proteins NCL and FBL in protection from genomic stress, which was further validated by the demonstration of stable complexes of endogenous Notch4 and the nucleolar proteins NCL and NPM (but not FBL) in immunoprecipitation analysis. Ablation of Notch4 but not Notch1 in the breast cancer cell lines MDA-MB-231 and Hs578T modulated sensitivity to genotoxic stress, which agreed with outcomes of overexpressing NIC4 and its role in modulating signaling cascades that confer protection from genomic damage. We further establish that canonical DNA repair proteins were key downstream intermediates.

Perturbation of the NoLS results in complete nucleolar exclusion in all cells examined, indicating that this sequence is necessary and sufficient for nucleolar localization of NIC4. NIC4_3RA-GFP, the nucleolar excluded form, failed to protect from genomic damage although *Hes* transcription (a nuclear function) or inhibition of apoptosis triggered by ER stress was unimpaired. Thus, despite the observed mobility in the nuclear and nucleolar pools, functions of the two pools are likely distinct, with nucleolar localization required specifically for NIC4 activity vis-à-vis protection from genomic damage. Notably, the closely related protein, NIC1, which also protects from genomic damage, does not require nucleolar localization, although its signaling, like NIC4, is independent of the canonical partner, RBPj-κ^34^. Since the NoLS in NIC1 includes Lysine and not Arginine (as in NIC4) residues, we speculate that nucleolar localization in NIC1 may be regulated by posttranslational modification resulting in a net reduction of overall positive charge. The acetylation of lysine residues in NIC1 has been reported in other contexts^35–37^; however, it remains to be established whether this modification regulates nucleolar localization of NIC1.

Why might nucleolar localization provide a survival advantage to cells? Based on our observations and the role of the nucleolus in maintenance of cellular homeostasis^38,39^, we speculate that nucleolar NIC4 association with Nucleolin and other proteins may play a role in maintaining the structural integrity of the nucleolus, especially in the context of genomic stress. This may stabilize the DNA repair machinery, also localized in the nucleolus, thereby enabling recovery of cells subjected to genotoxic stress, which is consistent with the differential susceptibility of breast cancer cells to genomic damage. Our data also suggest that signaling from Notch4 and Notch1 activate different pathways for protection as the molecular interactions of these proteins and ensuing signaling are distinct (ref. ^34^ and this work). Collectively, this study provides yet another example of how spatial regulation of the Notch family^14,16,17,40,41^ underpins signaling outcomes activated by these receptors.

## Materials and methods

### Cells

HEK293T (HEK), MDA-MB-231, Hs578T, BT-459, SUM149, and MCF7 cell lines were from ATCC (Manassas, VA, USA). HEK and MDA-MB-231 cells were maintained in Dulbecco's modified Eagle's medium (DMEM; GIBCO, Life Technologies USA) supplemented with 0.1% penicillin/streptomycin and 10% fetal bovine serum (Scientific Hyclone TM, Waltham, MA, USA) at 37 °C with 5% CO_2_. HCC1806, BT-549, Hs578T, and SUM149 cells were maintained in RPMI-1640 supplemented as above. Mycoplasma contamination in the cultures were tested using the MycoAlertTM Mycoplasma Detection Kit, Lonza (LT07-318).

### Reagents

5-FU (F6627), 4NQO (N8141), and Thapsigargin (T9033) were from Sigma-Aldrich (St. Louis, MO, USA). Etoposide (341205) was from Calbiochem-Merck Millipore (Darmstadt, Germany). Trizol and Superscript First Strand Synthesis System were from Invitrogen (CA, USA). SYBR™ Green Master Mix was from Thermo Scientific (CA, USA). Dharmafect-1 and siRNA to the scrambled control (D-0018010-10), Notch4 (L-011883-00), Notch1 (L-007771-00), RBPj-k (L-007772), Fibrillarin (L-011269), Nucleolin (L-003854), Rad50 (L-005232), Nbs1 (L-009641), and p53 (L-003329) were from Dharmacon (Lafayette, CO, USA). Antibodies to Notch4 (L5C5, 2423), Nucleolin (D4C70, 14574), and anti-rabbit Alexa 543 were from Cell Signaling Technology (MA, USA); NPM (FC82291, ab10530), Fibrillarin (EPR10823(B), ab166630), and Notch1 (mN1A, 128076) were from Abcam (Cambridge, MA). Antibody to actin (ACTN05, MS-1295-P), Normal Mouse IgG (NC-1255-P1), and Normal Rabbit IgG (NC-100-P1) were from Neomarker (Fremont, CA, USA).

### Plasmids

Human NIC4 was subcloned into pEGFP-N3 (BD Clontech, Mountain View, CA) between EcoRI and BamHI restriction sites to obtain NIC4-GFP using the following primers:

NIC4-EcoRI Forward: 5′-ATAGAATTCAATGCGGCGTCGAC-3′

NIC4-BamHI Reverse: 5′-TTAGGATCCTTTTTTACCCTCTC-3′

NoLS_NIC4 and NIC4_3RA mutants were generated using PCR-mediated mutations and addition of NIK (RKKRKKK) NoLS signal sequence to the former using the following primers:

NoLS_NIC4 Forward: 5′-TAGAATTCATGCGGAAGAAACGGAAGAAGAAGCGGCGTCGACGCCGAG-3′

NoLS_NIC 4 Reverse: 5′-AATGGATCCTTTTTTACCCTCTCCTCCTTG-3′

The following primers were used for the generation of the NIC43RA-GFP construct using PCR based site directed mutagenesis:

NIC4_3RA Forward: 5′-GCGCCTGCGACTCAGTCAGCTCCCCACCGACGCGCGCCCCCACTAGGCGAGGACAGC-3′

NIC4_3RA Reverse: 5′-CGCGCGTCGGTGGGGAGCTGACTGAGTCGCAGGCGCTCGAGTGAAACCAGGGGGCAGC-3′

mTagRFP-T-Fibrillarin-7 was a gift from Michael Davidson (Addgene plasmid #58016), GFP-Nucleolin from Michael Kastan (Addgene plasmid #28176), and human Bcl-xL-GFP plasmid from Richard J. Youle (National Institutes of Health, Bethesda, MD). Construct sequences were verified by automated Sanger sequencing conducted in-house.

### Transfections

HEK cells grown in flasks were trypsinized and seeded at a density of 0.25 × 10^6^ cells in (tissue culture grade) 35-mm dishes (Greiner Bio-one, Kremsmünster, Austria). In all, 100 nM siRNA or plasmids at the indicated concentrations were transfected using Dharmafect or Liofectamine-2000 as per the manufacturer’s instructions when cultures were 50–60% confluent (24 h post-plating). Cells transfected with siRNA were incubated for 24–26 h before being harvested by trypsinization and re-plated before transfections (with Lipofectamine 2000), 24 h after the last re-plating. Twenty-four hours after transfection, cells were treated with chemicals as described below. MDA-MB-231 and Hs578T cells were plated at 0.05–0.06 × 10^6^ cells per well in 24-well plates for transfections the next day, when cells reached 60–70% confluency. siRNA was transfected using RNAi MAX (Invitrogen, USA), following the manufacturer’s instructions. Plasmids were transfected using Lipofectamine 2000 or Lipofectamine-LTX at the following concentrations: NIC4-GFP (2 µg), pEGFP-N3 (1 µg), Bcl-xL GFP (2 µg); in MDA-MB-231 cells, NIC4-GFP (1.5 µg) and pEGFP-N3 (0.5 µg). Total DNA transfected in the different transfection groups was equalized with pcDNA3. Silencing was estimated by analyzing the transcript levels in cells transfected with control or gene-specific siRNA. Forty-eight hours post siRNA transfection, 0.5 × 10^6^ cells were lysed using TRIzol (Invitrogen) followed by RNA isolation as per the manufacturer’s instructions. Two micrograms of RNA was used for cDNA synthesis using the Superscript First Strand cDNA Synthesis Kit and real-time PCR was set-up using gene-specific primers.

### Induction of apoptosis and assays for cellular damage

To induce apoptosis, 24 h post-transfection cells were treated with etoposide (10 µM) or 5-FU (10 µM) or 4NQO (5 µM) for 48 h in serum-free DMEM (HEK) or 2.5% serum containing DMEM (MDA-MB-231). Cells were treated with Thapsigargin (10 µM) for 20 h in serum-free DMEM. Cells were harvested and stained with Hoechst 33342 (1 μg/ml), and samples were scored for nuclear damage in GFP-positive cells using fluorescent microscopy (Olympus BX-60). Samples were blinded for the experimenter and approximately 200 cells in 5 random fields were scored for apoptotic damage.

### Immunostaining

Cells were plated at 0.1 × 10^6^ cells in cut confocal dishes and cultured for 48 h to adhere and increase in number. The monolayer was fixed with 2% paraformaldehyde (PFA; freshly reconstituted) and incubated in the dark for 20 min at room temperature. Dishes were permeabilized using 0.2% Triton-X 100 for 10 min at ambient temperature and blocked in freshly made buffer (5% normal goat serum, 0.3% Triton-X 100) for 1 h at ambient temperature. Samples were treated with primary antibody, added at the indicated dilutions in 5% bovine serum albumin–phosphate-buffered saline (PBS), Nucleolin (1:100), N4 (1:100), and N1 (1:100) for 2.5 h at ambient temperature. Samples were washed 2× in PBS and secondary fluorescence-conjugated antibody was added and samples were incubated for 1 h, protected from light at ambient temperature. Samples were washed 2× with PBS and counterstained with Hoechst 33342, and seven random fields were imaged using Olympus FV3000 confocal microscope (×63 NA 1.35 oil-immersion objectives).

### Co-localization analysis

Cells transfected with NIC4-GFP and Fibrillarin-RFP (1 µg) were cultured for 24 h post-transfection and then fixed with 2% PFA. Images were acquired using Olympus FV3000 confocal microscope (×63 NA 1.35 oil-immersion objectives). Co-localization of NIC1-GFP or NIC4-GFP with Fibrillarin-RFP was quantified using co-localization threshold plugin after minimizing background in the Fiji ImageJ software. Manders correlation coefficient is proportional to the fraction of fluorescence intensity of one channel that co-localizes with the other channel and ranges from 0 (no co-localization) to 1 (maximum co-localization).

### Real-time PCR

Total RNA was isolated using TRIzol reagent according to the manufacturer’s instructions, and concentration was determined using the Nanodrop 2000 (Thermo Scientific). Two micrograms of total RNA was used for cDNA synthesis using SuperScript First-Strand Synthesis System. Real-time PCR was performed using Maxima™ SYBR Green qPCR Master Mix and Bio-Rad CFX96 Touch™ Real-Time PCR Detection System. Relative change in gene expression was calculated using 2^−ΔΔCt^ method using glyceraldehyde 3-phosphate dehydrogenase (GAPDH) as the reference gene. The primers used for reverse transcription PCR are as follows:

Gene: Forward (5’–3’); Reverse (5’–3’)

GAPDH: TGCACCACCAACTGCTTAGC; GGCATGGACTGTGGTCATGAG

FBL: TGGACCAGATCCACATCAAA; GACTAGACCATCCGGACCAA

NCL: CCAGCCATCCAAAACTCTGT; TAACTATCCTTGCCCGAACG

RAD50: GGGTTTCCAAGGCTGTGCTA; TCTGACGTACCTGCCGAAGT

NBN: CACTCACCTTGTCATGGTATCAG; CTGCTTCTTGGACTCAACTGC

HES5: CCGGTGGTGGAGAAGATGCG; GCGACGAAGGCTTTGCTGTG

RBPJK: AACAAATGGAACGCGATGGTT; GGCTGTGCAATAGTTCTTTCCTT

Notch4: GCGGAGGCAGGGTCTCAACGGATG; AGGAGGCGGGATCGGAATGT

Notch1: TCCACCAGTTTGAATGGTCA; AGCTCATCATCTGGGACAGG

### FRAP and FLIP analysis

Cells were plated and transfected on sterile coverslips fixed in Petri dishes to allow for confocal imaging and analysis of cells without trypsinization. Cells were co-transfected with NIC4-GFP (0.5 µg) and Fibrillarin-RFP (0.5 µg) 18–24 h prior to imaging using Olympus FV3000 (oil immersion objective, ×63 1.35 NA). The FRAP module was used to photo-bleach and acquire images at 2-s intervals immediately after photo-bleaching the region of interest (ROI, white arrowhead in images). Fluorescence recovery was quantified after correcting for photo-bleaching using the cellSens software.

For FLIP analysis, cells were transfected with NIC4-GFP (0.5 µg) and Fibrillarin-RFP (0.5 µg) and cultured for 24 h. FLIP analysis was performed on Olympus FV3000 at ×60 oil objective with 37 °C stage incubator. ROI was drawn around nucleus excluding nucleolus and the ROI photo-bleached for 700 ms with 60% of laser (488 or 561). Time-lapse images were acquired at 2-s intervals before and after bleaching. Fluorescence intensities of NIC4-GFP and Fibrillarin-RFP in the ROI restricted to the nucleolus were quantified using the Fiji ImageJ software.

### Immunoprecipitation and western blotting

In all, 3 × 10^6^ MDA-MB-231 cells were treated with etoposide (10 μM) in serum-free DMEM for 6 h before immunoprecipitation. Cells were lysed using RIPA buffer containing 1% NP-40, 50 mM Tris, 1 mM NaCl, and 1 mM EDTA and supplemented with aprotinin, leupeptin, and pepstatin (2 μg/ml each), 10 μM MG132, 1 mM phenylmethylsulfonyl fluoride, 1 mM NaF, and 1 mM Na_3_VO_4_. Cell lysates were centrifuged at 5000 rpm for 5 min to remove cell debris, and supernatants were incubated with antibody against Notch4 (1:50), Nucleolin (1:50), Normal Mouse IgG (1.5 µg), or Normal Rabbit IgG (1.5 µg) for 2 h at 4 °C on rotational mixer. After 2 h, Sepharose G/A plus bead slurry was added to the cell lysate/antibody mix and incubated for 2 h at 4 °C on a rotational mixer. Beads bound to immune-complexes were washed three times with ice-cold PBS by gentle centrifugation, and bead pellets were re-suspended in sodium dodecyl sulfate (SDS) lysis buffer containing protease inhibitors as mentioned above and boiled for 10 min before analyzing by western blotting. Immune-complexes were resolved on a 10% SDS–polyacrylamide gel and transferred to a nitrocellulose membrane (GE Healthcare). Membranes were blocked with 5% skimmed milk for 1 h at ambient temperature and incubated with primary antibodies at 4 °C overnight. The membranes were washed thrice with Tris-buffered saline–Tween 20 (TBST), followed by incubation with horseradish peroxidase–conjugated secondary antibody (CST, 1:1000 dilution) for 1 h at ambient temperature. Membranes were developed with SuperSignal West Dura substrate (Thermo Scientific), and images were acquired using iBright FL1000 (Thermo Scientific). For western blot analysis, primary antibodies were diluted at 1:1000 in 5% nonfat dried milk in TBST.

### Statistical analysis

Data are represented as mean ± standard deviation (Mean ± SD) for two or three independent experiments. Statistical significance was measured using two-tailed Student’s *t* test and *p* values < 0.01 were considered to be statistically significant. Data plotted for FRAP and FLIP analysis are mean ± SD from two experiments.

## Supplementary information

Supplementary Material
