# Supplementary material for: Nucleolar localization of the Notch4 intracellular domain underpins its regulation of the cellular response to genotoxic stressors
Source: Cell Death Discov. 2020 Feb 18;6:7. doi: 10.1038/s41420-020-0242-y (PMC7029026; doi:10.1038/s41420-020-0242-y)
Supplement: Supplementary file 1 — Supplementary Material [file 41420_2020_242_MOESM1_ESM.docx]

**Supplementary Material**

**Nucleolar localization of the Notch4 intracellular domain underpins its regulation of the cellular response to genotoxic stressors**

Neetu Saini^1,2^ and Apurva Sarin^1^

^1^Institute for Stem Cell Science & Regenerative Medicine (inStem), Bellary Road, Bengaluru, Karnataka, India; ^2^Department of Biology, **Manipal Academy of Higher Education**, Manipal, India.

**This file includes:**

Supplementary Figures 1-5

Supplementary Figures legend 1-5

**Supplementary Figure 1**

**
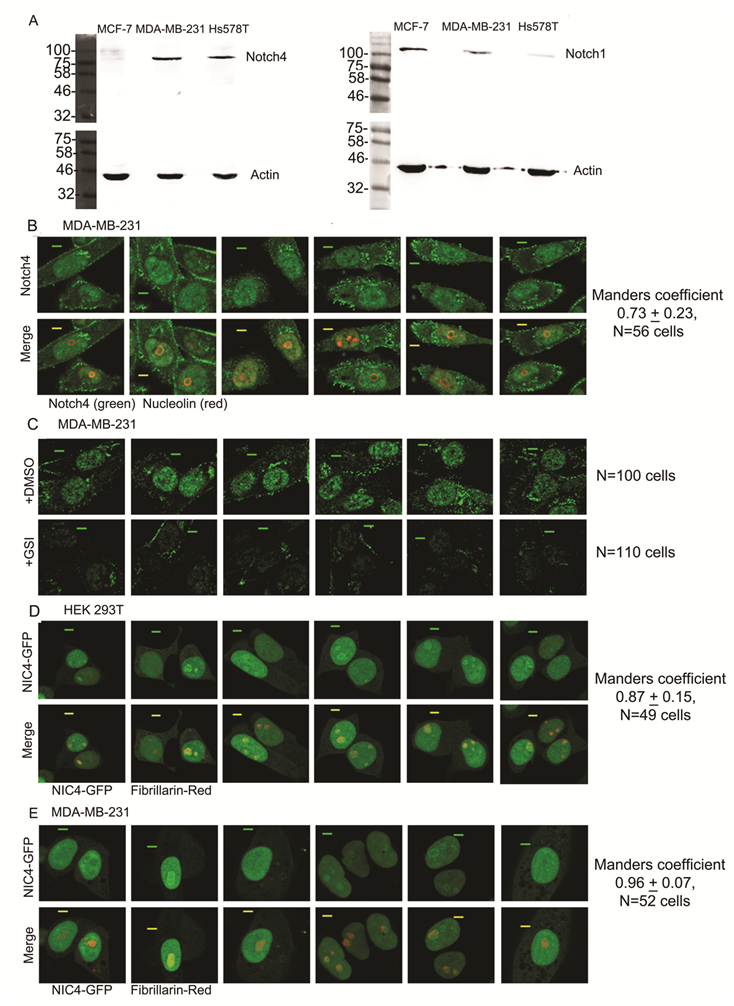
**

**Supplementary Figure 1.** (**A**) Immunoblots of lysates of breast cancer cell lines probed for Notch4 and Notch1. (**B**) Representative confocal resolution images of MDA-MB-231 cells, immunostained with an antibody against Notch4 (green, upper panel) and merge (lower panel) of staining for Notch4 and Nucleolin (red). (**C**) Representative confocal resolution images of MDA-MB-231 cells treated with GSI (lower panel) or vehicle control (DMSO, upper panel) as described in methods and immunostained with an antibody against Notch4. (**D** and **E**) HEK (**D**) or MDA-MB-231 (**E**) cells co-transfected with NIC4-GFP and Fibrillarin-RFP and cultured for 24 h. Cells were fixed and confocal images were acquired. Images of NIC4-GFP (upper panel) and the merge of the same cells co-expressing Fibrillarin-RFP (lower panel) are shown. scale bar: 5µm.

**Supplementary Figure 2**

**
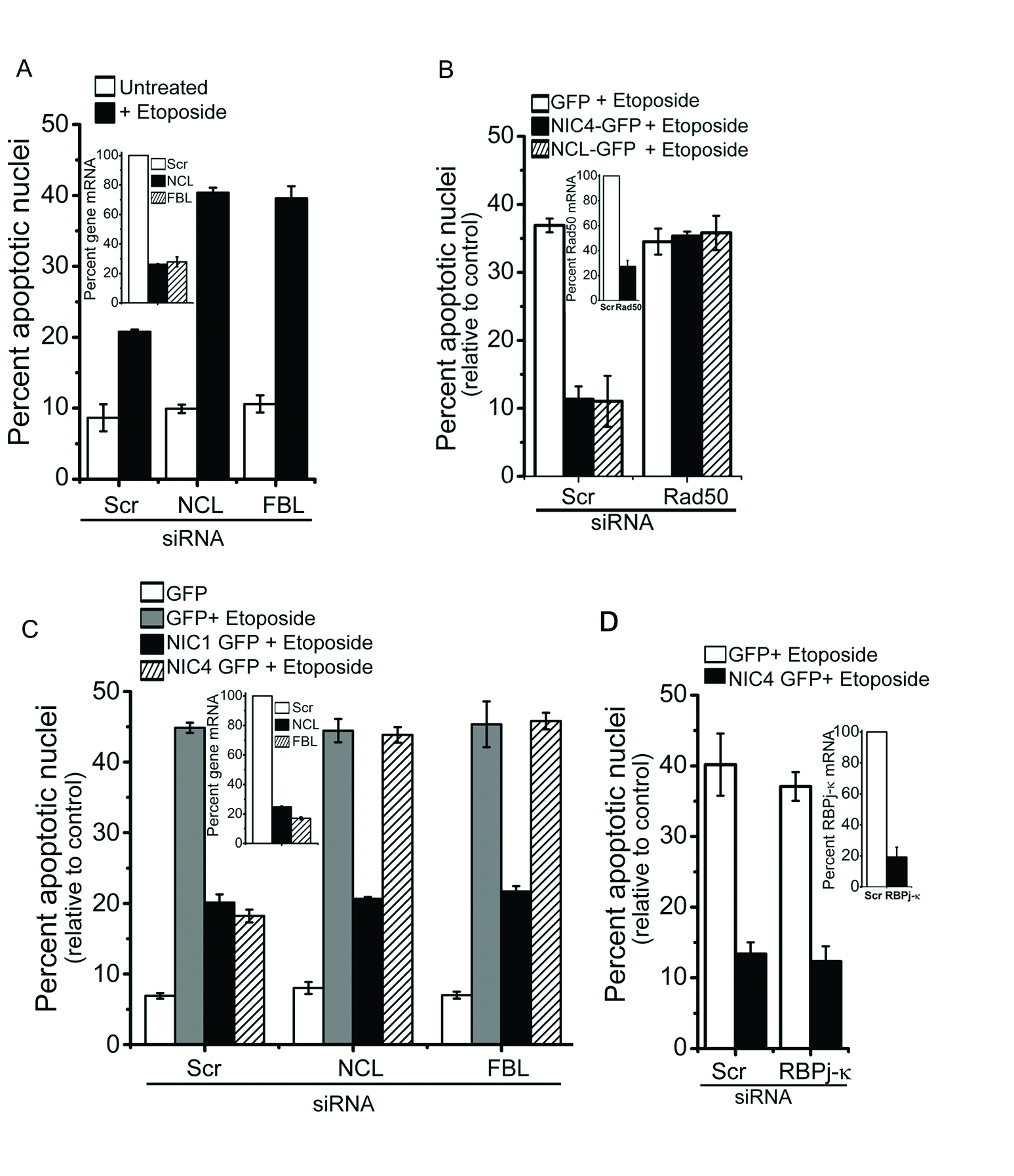
**

**Supplementary Figure 2.** (**A**) Induction of apoptotic nuclear damage in MDA-MB-231 cells pre-treated with indicated siRNA and treated with 10μM etoposide for 24 h. (**B**) Induction of etoposide induced apoptotic damage, measured at 48 h in cells expressing the indicated plasmids, following prior treatment with siRNA to Rad50 or scrambled control. (**C**) HEK cells were pre-treated with indicated siRNA, transfected with GFP, or NIC1-GFP or NIC4-GFP and assessed for induction of apoptotic damage after 48 h treatment with 10μM etoposide. (**D**) Induction of etoposide induced apoptotic damage in HEK cells, measured at 48 h in cells transfected with GFP or NIC4-GFP, following prior treatment with siRNA to RBPj-κ or scrambled control. In all panels, insets show mRNA levels in the different groups. The data plotted are mean ± SD of two independent experiments in panels **A-C** and three independent experiments in panel **D**.

**Supplementary Figure 3**


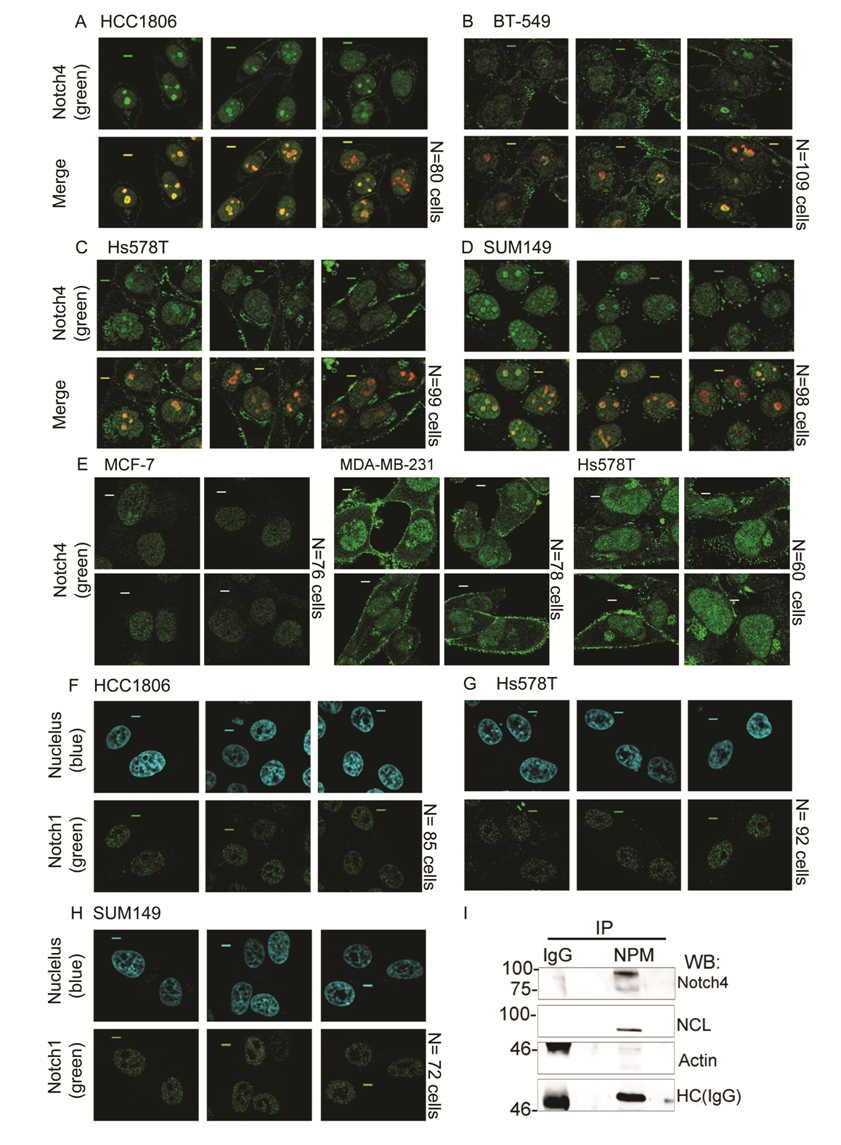


**Supplementary Figure 3.** (**A-E**) Confocal images of breast cancer lines immunostained for Notch4 (Green, upper panel in each case) and the merge in cells stained for Nucleolin (Red, lower panel in each case). (**F-H**) Confocal images of breast cancer cells immunostained with Notch1 (green) and counterstained with Hoechst 33342 (blue). (**I**) MDA-MB-231 cells treated with etoposide for 6 h were lysed and subject to immunoprecipitation with antibody to NPM and IgG (isotype control). Immunoprecipitates were analysed by western blotting for Notch4, NCL, Actin and IgG. Immunoblots are representative of two independent experiments. Scale bar: 5μm.

**Supplementary Figure 4**


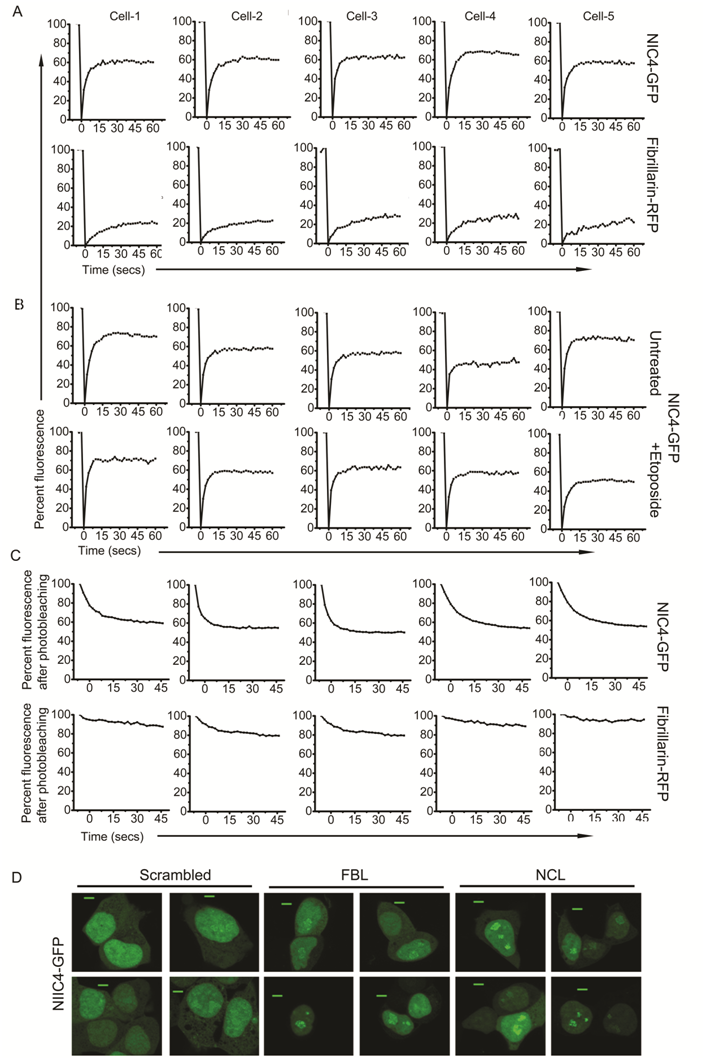


**Supplementary Figure 4.** (**A**) Quantitation of fluorescence recovery after photo-bleaching in HEK cells transfected with NIC4-GFP (0.5μg) (Top) and Fibrillarin-RFP (0.5µg) (Bottom) and cultured for 24 h. (**B**) Cells expressing NIC4-GFP (0.5µg) for 24 h were cultured without (top) or with (bottom) etoposide (10μM) for 6 h and fluorescence recovery of GFP after photo-bleaching the nucleolus was measured in cells expressing NIC4-GFP. (**C**) Fluorescence loss of NIC4-GFP (top) or Fibrillarin-RFP (bottom) in the nucleolus after photo-bleaching the nucleoplasm in cells expressing NIC4-GFP and Fibrillarin-RFP. (**D**) Representative confocal images of cells expressing NIC4-GFP following pre-treatment for 48 h with scrambled control or FBL or NCL siRNA (n>30 in three independent experiments); scale bar: 5μm.

**Supplementary Figure 5**


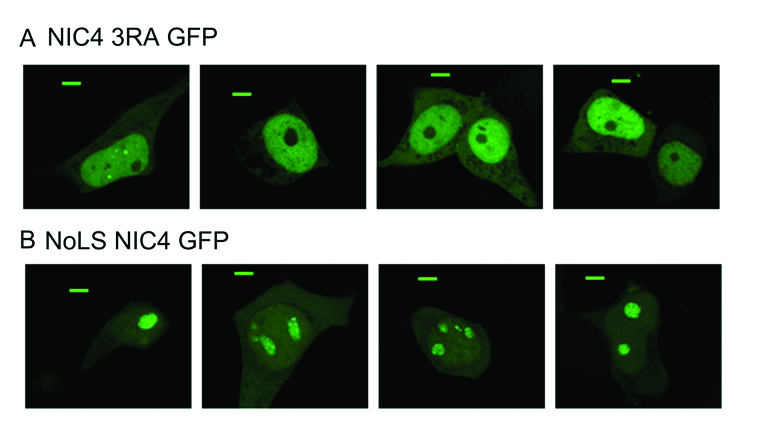


**Supplementary Figure 5.** (**A** and **B**) Representative confocal images of live HEK cells expressing NIC4_3RA GFP (**A**) or NoLS NIC4 GFP (**B**), imaged 24 h post-transfection (n>20 cells). scale bar: 5μm.
